# Role of Concurrent Ultrasound Surveillance of Sentinel Node-Positive Node Fields in Melanoma Patients Having Routine Cross-Sectional Imaging

**DOI:** 10.1245/s10434-023-14526-9

**Published:** 2023-11-15

**Authors:** Caroline A. Gjorup, Rachel Woodford, Isabel Li, Matteo S. Carlino, Sydney Ch’ng, David Chung, Edward Hsiao, Serigne N. Lo, Kevin London, Georgina V. Long, Alexander M. Menzies, Omgo E. Nieweg, Thomas E. Pennington, Michael A. Rtshiladze, Robyn P. M. Saw, Richard A. Scolyer, Kerwin F. Shannon, Andrew J. Spillane, Jonathan R. Stretch, John F. Thompson, Alexander H. R. Varey, Alexander C. J. van Akkooi

**Affiliations:** 1 Melanoma Institute Australia, The University of Sydney, Sydney, NSW Australia; 2 NHMRC Clinical Trials Centre, The University of Sydney, Sydney, Australia; 3 Department of Medical Oncology, Westmead Hospital, Westmead, Australia; 4 Faculty of Medicine and Health, The University of Sydney, Sydney, Australia; 5 Department of Medical Oncology, Blacktown Hospital, Blacktown, Australia; 6 Department of Plastic Surgery, Royal Prince Alfred Hospital, Camperdown, Australia; 7 Chris O’Brien Lifehouse Cancer Centre, Head and Neck Service, Camperdown, Australia; 8 Alfred Nuclear Medicine and Ultrasound, Newtown, Australia; 9 Department of Nuclear Medicine, The Children’s Hospital at Westmead, Westmead, Australia; 10 Mater Imaging, Mater Hospital, North Sydney, Australia; 11 Department of Medical Oncology, Royal North Shore Hospital, St Leonards, Australia; 12 Department of Medical Oncology, Mater Hospital, North Sydney, Australia; 13 Charles Perkin Centre, The University of Sydney, Sydney, Australia; 14 Department of Melanoma and Surgical Oncology, Royal Prince Alfred Hospital, Camperdown, Australia; 15 Department of Surgery, Mater Hospital, North Sydney, Australia; 16 Tissue Pathology and Diagnostic Oncology, Royal Prince Alfred Hospital, Camperdown, Australia; 17 NSW Health Pathology, Sydney, Australia; 18 Breast and Melanoma Surgery Unit, Royal North Shore Hospital, St Leonards, Australia; 19 Department of Plastic Surgery, Westmead Hospital, Westmead, Australia

**Keywords:** Melanoma, Sentinel node (SN), Sentinel lymph node (SLN), Sentinel lymph node biopsy (SLNB), Stage III, Imaging, Ultrasound, CT, PET/CT, Surveillance, Recurrence, Nodal, Adjuvant therapy, Diagnosis

## Abstract

**Purpose:**

In sentinel node-positive (SN+ve) melanoma patients, active surveillance with regular ultrasound examination of the node field has become standard, rather than completion lymph node dissection (CLND). A proportion of these patients now receive adjuvant systemic therapy and have routine cross-sectional imaging (computed tomography [CT] or positron emission tomography [PET]/CT). The role of concurrent ultrasound (US) surveillance in these patients is unclear. The purpose of our study was to describe the modality of detection of nodal recurrence in SN+ve node fields.

**Methods:**

SN+ve melanoma patients who did not undergo CLND treated at a single institution from January 1, 2016 to December 31, 2020 were included.

**Results:**

A total of 225 SN+ve patients with a median follow-up of 23 months were included. Of these, 119 (53%) received adjuvant systemic therapy. Eighty (36%) developed a recurrence at any site; 24 (11%) recurred first in the SN+ve field, of which 12 (5%) were confirmed node field recurrence only at 2 months follow-up. The nodal recurrences were first detected by ultrasound in seven (3%), CT in seven (3%), and PET/CT in seven (3%) patients. All nodal recurrences evident on US were also evident on PET/CT and vice versa.

**Conclusions:**

The high rate of recurrences outside the node field and the identification of all US-detected nodal recurrences on concurrent cross-sectional imaging modalities suggest that routine concurrent ultrasound surveillance of the node-positive field may be unnecessary for SN+ve melanoma patients having routine cross-sectional imaging.

**Supplementary Information:**

The online version contains supplementary material available at 10.1245/s10434-023-14526-9.

Contemporary surgical management of primary cutaneous melanomas in patients at high risk of regional lymph node metastasis involves wide local excision and sentinel node (SN) biopsy (SNB). If a SN is found to contain metastatic melanoma, active surveillance of the node field is currently widely accepted. Previously, completion lymph node dissection (CLND) was recommended for SN-positive (SN+ve) melanoma patients. However, two seminal randomized trials, MSLT-II and DeCOG-SLT, failed to demonstrate a melanoma-specific survival (MSS) advantage from immediate CLND in SN+ve patients compared with nodal surveillance and delayed therapeutic lymph node dissection (TLND) for node field recurrence only.^1,2^ Where CLND was omitted in these trials, ultrasound surveillance of the SN+ve node field was mandated.^1,2^ Disease-free survival was improved for the immediate CLND group, but this came at the cost of a significantly higher risk of morbidity. Thus, CLND is no longer performed routinely.

Since these trials commenced recruiting patients in 2004 and 2006 respectively, systemic therapy options for melanoma patients have been revolutionized. Early adjuvant systemic therapy trials mandated CLND for SN+ve patients; however, this is no longer the case. Many of these patients are now offered adjuvant systemic therapy with either immune checkpoint inhibitors or BRAF/MEK inhibitors, both of which have been shown to significantly improve recurrence-free survival (RFS) compared with placebo (or ipilimumab).^3–5^

In patients who receive adjuvant systemic therapy, frequent cross-sectional imaging is usually performed, commonly at 3-monthly or 4-monthly intervals for the first 2 years and with decreasing frequency thereafter. Some patients who only have active surveillance undergo cross-sectional imaging at the same intervals as those receiving adjuvant therapy. Adjuvant therapy trials did not mandate ultrasound surveillance of the SN+ve field, despite US previously having been shown to be superior to computed tomography [CT], positron emission tomography [PET], and PET/CT for the early detection of regional lymph node metastases.^3–6^ Consequently, routine US often is not part of the surveillance imaging schedule, particularly when medical oncologists are undertaking the follow-up. Ultrasound is still commonly performed in routine practice for regional node surveillance of patients not receiving systemic adjuvant therapy and undergoing follow-up with their surgeon and/or referring dermatologist or primary care physician. This is frequently in conjunction with less frequent whole-body cross-sectional imaging.

Whether there is still a place for US surveillance of the regional lymph nodes of SN+ve patients in the modern era is unclear, particularly given the frequent use of whole-body cross-sectional imaging. To address this question, the purpose of this study was to describe the sites of recurrence and modality of their detection in SN+ve melanoma patients.

## Methods

### Study Design

For this retrospective study, data were extracted from a prospectively-maintained database for patients with primary cutaneous melanoma and a positive SN treated at a large Australian melanoma treatment centre (Melanoma Institute Australia [MIA]) from January 1, 2016 to December 31, 2020 and who did not have CLND. Patients were included if they had a clear baseline scan at the time of their SNB or if they did not have a baseline scan, a minimum 2-month interval from the date of SNB to the detection of nodal recurrence in the SN field. Exclusion criteria were a previous or subsequent higher-stage melanoma or concurrent in-transit metastases. Written consent for the use of their data had been obtained from all patients. The study was approved by the MIA Research Committee (MIA2022/447) under Human Research Ethics Committee (HREC) Sydney Local Health District Protocol No X15-0311 & 2019/ETH06854.

### Outcomes

The following variables were collected for the whole cohort: sex, age at time of SNB, melanoma subtype, location of primary tumor (head or neck, upper limb, lower limb, trunk), Breslow thickness (mm), ulceration status, mitotic rate, microsatellites, extracapsular extension, site(s) and if more than one site of excised SN and total number of excised LNs (SNs and non-SNs), total number of positive SNs, AJCC stage (8^th^ edition) at time of diagnosis, adjuvant therapy (and type) if administered, duration of follow-up, and status at last follow-up.

The primary endpoint was modality of detection (patient, clinician, US, CT, or PET/CT) of nodal recurrence, as the first site of recurrence, in a node field from which a positive SN had previously been removed. Nodal recurrence was defined as node field recurrence only or node field and other site(s) (e.g., in-transit or distant metastasis), with the latter defined as any further recurrence detected within 2 months of the nodal recurrence. Node field recurrence and other sites was defined as radiologically evident metastatic disease with or without biopsy confirmation.

Secondary endpoints included whether imaging performed within 2 months of the nodal recurrence demonstrated the node recurrence, days between the scans (US, PET/CT, and/or CT), time to nodal recurrence (from SNB to nodal recurrence in SN field), adjuvant therapy at the time of nodal recurrence, and if recurrence occurred while on adjuvant therapy whether this led to a change in treatment. If the patient recurred in the node field as well as elsewhere concurrently, the burden of disease was recorded as oligometastatic disease (1–3 site(s)) or high-volume disease (≥4 sites), and sites of disease were documented.

Details of surgical treatment of nodal recurrence were collected, including whether this was performed in patients with node field recurrence only or node field and other site(s) recurrences, type of surgery (selective excision of involved nodes or TLND), number of involved nodes, largest diameter of the excised nodal metastasis, and presence or absence of extracapsular extension. The maximum diameter of each excised nodal metastasis was further stratified according to the imaging modality by which it was first detected. Lastly, the size of the nodal metastasis (the short axis of the lymph node metastasis), measured on the imaging modality, which detected the nodal recurrence, was documented.

### Descriptive Analysis

Key summary statistics were derived for patients’ characteristics and clinicopathologic features. All percentages were calculated as relative to the whole cohort. To explore associations between each variable and different types of recurrences, *P*-values were obtained from Kruskal-Wallis rank sum tests for continuous variables and Fisher’s exact tests for categorical variables. Missing values were not considered when computing the Fisher’s exact test.

## Results

### Patient Population, Imaging, and Follow-Up

Between 2016 and 2020, 225 SN+ve patients in whom CLND was omitted underwent surveillance as recommended by their surgeon and/or medical oncologist (Table 1). Typically, the surveillance included clinical examination and scans. The scans (US, PET/CT, and/or CT), and their frequency differed, depending on perceived risk of recurrence, individual surgeon, and/or medical oncologist preference or clinical trial protocol. Of the 225 SN+ve patients, 119 (53%) received adjuvant systemic therapy; 27 (12%), 35 (16%), 55 (24%), and two (1%) received adjuvant therapy for stage IIIA, IIIB, IIIC, and IIID disease, respectively. The median follow-up was 23 months (interquartile range [IQR] 12–34).

**Table 1:** Patient and disease characteristics of sentinel node positive patients. All percentages are given of the whole cohort, *N* = 225

	Total (*N* = 225)	Node field recurrence only (*n* = 24)	Recurrence at any site except node field (*n* = 56)	No recurrence (*n* = 145)	*P* value
Sex					0.100
Male	138 (61%)	10 (4%)	35 (16%)	93 (41%)	
Female	87 (39%)	14 (6%)	21 (9%)	52 (23%)	
Age					< 0.001
Mean (SD)	59 (15)	62 (14)	64 (15)	56 (15)	
Median (IQR)	61 (48–71)	63 (53–74)	68 (59–73)	57 (45–67)	
Melanoma subtype					0.281
Lentigo maligna	7 (3%)	0 (0%)	3 (1%)	4 (2%)	
Superficial spreading	95 (42%)	10 (4%)	19 (8%)	66 (29%)	
Nodular	72 (32%)	5 (2%)	23 (10%)	44 (20%)	
Acral	14 (6%)	4 (2%)	2 (1%)	8 (4%)	
Other	37 (16%)	5 (2%)	9 (4%)	23 (10%)	
Primary melanoma					0.969
Head & neck	39 (17%)	3 (1%)	10 (4%)	26 (%)	
Trunk	92 (41%)	10 (4%)	22 (10%)	60 (27%)	
Upper extremity	29 (13%)	2 (1%)	8 (4%)	19 (8%)	
Lower extremity	65 (29%)	9 (4%)	16 (7%)	40 (18%)	
Breslow thickness (mm)					< 0.001
Mean (SD)	3.7 (3.3)	4.4 (3.1)	4.9 (3.2)	3.2 (3.3)	
Median (IQR)	2.8 (1.7–4.7)	3.4 (2.4–4.7)	4.4 (2.2–7.8)	2.4 (1.5–3.6)	
Ulceration					0.006
No	116 (52%)	8 (4%)	22 (10%)	86 (38%)	
Yes	106 (47%)	16 (7%)	33 (15%)	57 (25%)	
missing	3 (1%)	0 (0%)	1 (0.5%)	2 (1%)	
Mitoses (mm^2^)					0.001
mean (SD)	7.5 (7.9)	10.6 (10.9)	10.1 (9.7)	5.9 (6.0)	
Median (IQR)	5.0 (2.0–10.0)	7 (3.0–12.0)	8.0 (3.8–14.0)	4 (2.0–7.0)	
0	6 (3%)	0 (0%)	1 (0.5%)	5 (2%)	
1-2	52 (23%)	5 (2%)	8 (4%)	39 (17%)	
3-5	59 (26%)	2 (1%)	11 (5%)	46 (20%)	
6+	105 (47%)	16 (7%)	36 (16%)	53 (24%)	
Missing	3 (1%)	1 (0.5%)	0 (0%)	2 (1%)	
Microsatellites					0.028
No	187 (83%)	22 (10%)	43 (19%)	122 (54%)	
Yes	20 (9%)	2 (1%)	10 (4%)	8 (4%)	
missing	18 (8%)	0 (0%)	3 (1%)	15 (10%)	
Extracapsular extension					0.244
No	212 (94%)	21 (9%)	54 (24%)	137 (61%)	
Yes	13 (6%)	3 (1%)	2 (1%)	8 (4%)	
Site of sentinel node					0.673
Head & neck	37 (16%)	3 (1%)	9 (4%)	25 (11%)	
Axilla	83 (37%)	8 (4%)	20 (9%)	55 (24%)	
Groin	67 (30%)	10 (4%)	13 (6%)	44 (20%)	
More than one site	34 (15%)	3 (1%)	13 (6%)	18 (8%)	
Other	4 (2%)	0 (0%)	1 (0.5%)	3 (1%)	
Total excised SN					0.261
Mean (SD)	3.0 (2.2)	2.4 (1.6)	3.1 (2.4)	3.0 (2.2)	
Median (IQR)	2.0 (2.0–4.0)	2.0 (1.0–3.2)	3.0 (2.0–3.0)	2.0 (2.0–4.0)	
Total positive SN					0.308
Mean (SD)	1.3 (0.6)	1.4 (0.7)	1.4 (0.8)	1.3 (0.6)	
Median (IQR)	1.0 (1.0–2.0)	1.0 (1.0–2.0)	1.0 (1.0–2.0)	1.0 (1.0–1.0)	
Total excised nodes					0.165
Mean (SD)	3.3 (2.9)	2.5 (1.8)	3.4 (2.6)	3.3 (3.2)	
Median (IQR)	2.0 (2.0–4.0)	2.0 (1.0–3.2)	3.0 (2.0–4.0)	2.0 (2.0–4.0)	
Total positive nodes					0.145
Mean (SD)	1.4 (0.7)	1.5 (0.7)	1.5 (0.8)	1.3 (0.6)	
Median (IQR)	1.0 (1.0–2.0)	1.0 (1.0–2.0)	1.0 (1.0–2.0)	1.0 (1.0–1.0)	
AJCC stage 8th edition					0.002
IIIA	60 (27%)	2 (1%)	8 (4%)	50 (22%)	
IIIB	48 (21%)	5 (2%)	10 (4%)	33 (15%)	
IIIC	113 (50%)	16 (7%)	37 (16%)	60 (27%)	
IIID	4 (2%)	1 (0.5%)	1 (0.5%)	2 (1%)	
Adjuvant therapy*					0.115
Nivolumab/Pembrolizumab	114 (51%)	10 (4%)	22 (10%)	82 (36%)	
Targeted	5 (2%)	0 (0%)	1 (0.5%)	4 (2%)	
None	106 (47%)	14 (6%)	33 (15%)	59 (26%)	
Duration of follow-up (months)					0.001
Median (IQR)	23 (12–34)	23 (14–38)	28 (17–39)	21 (11–30)	
Status at last follow-up					< 0.001
Alive with melanoma	30 (13%)	8 (4%)	22 (10%)	0 (0%)	
Alive without melanoma	166 (74%)	10 (4%)	17 (8%)	139 (62%)	
Dead with melanoma	20 (9%)	5 (2%)	15 (7%)	0 (0%)	
Dead without melanoma	9 (4%)	1 (0.5%)	2 (1%)	6 (3%)	

*SD* standard deviation, *IQR* interquartile range, *AJCC* American Joint Committee on Cancer

*P* values were obtained from Kruskal–Wallis rank-sum tests for numerical variables and Fisher exact tests for categorical variables. In all Fisher exact tests, missing values were excluded

*27 (12%), 35 (16%), 55 (24%), and 2 (1%) received adjuvant therapy for stage IIIA, IIIB, IIIC, and IIID, respectively

### Recurrence

At last follow-up, 12 patients (5%) had developed an initial first recurrence only in the draining lymph node field, 12 patients (5%) had recurred at multiple sites, including the SN+ve field, 56 (25%) had recurred only outside the node field, and 145 (64%) were recurrence-free. The descriptive tests showed that greater age, greater Breslow thickness, presence of ulceration, higher mitotic rate, presence of microsatellites, and higher AJCC stage were associated with recurrence (Table 1). The median time to nodal recurrence was 10 months (IQR 5–15; Table 2). Six (3%) of the 24 nodal recurrences were diagnosed whilst the patient was receiving adjuvant therapy.

**Table 2:** Detection of nodal recurrence in the SN+ve field

	Nodal recurrence, *n* = 24 (percentages are of the whole cohort of SN+ patients, *N* = 225)
Mode of detection of nodal recurrence	
Patient	1 (0.5%)
Clinician	2 (1%)
US	7 (3%)
CT	7 (3%)
PET/CT	7 (3%)
Disease at the time of detection of nodal recurrence	
Node field recurrence only	12 (5%)
Concurrent node field and other site(s) (e.g., in-transit or distant metastasis)	12 (5%)
Imaging performed within 2 months of nodal recurrence	
CT + US + PET/CT	9 (4%)
CT + PET/CT	2 (1%)
US + PET/CT	8 (4%)
Single scan*	4 (2%)
No scan performed	1 (0.5%)
Nodal recurrence evident on	
CT + US + PET/CT**	8 (4%)
CT + PET/CT	2 (1%)
US + PET/CT	8 (4%)
Scans interval (days)	
Median (IQR)	18 (10–24)
Range	0–44
Time to nodal recurrence (months)	
Median (IQR)	10 (5, 15)
Range	3–37
Adjuvant therapy at time of nodal recurrence	
Yes	6 (3%)
Recurred after adjuvant therapy	12 (5%)
No previous adjuvant therapy	6 (3%)
If on adjuvant therapy at the time of nodal recurrence, change hereof	
Yes	6 (3%)
Not applicable	18 (8%)

*IQR* interquartile range, *US* ultrasonography, *CT* computed tomography, *FDG-PET/CT* fluorodeoxyglucose positron emission tomography-CT, *TLND* therapeutic lymph node dissection

*Single scan, US, CT, or FDG-PET/CT

**One patient had nodal recurrence evident on US and FDG-PET/CT, which was not detected on CT

### Detection of Nodal Recurrence

Nodal metastases were detected as a first site of recurrence in 24 patients. These were first detected by imaging in 21 patients (9%), by the patient in one (0.5%), and by the clinician at the physical examination in two (1%). The imaging modality first detecting the nodal recurrence was ultrasound in seven patients (3%), CT in seven patients (3%), and PET/CT in seven patients (3%). No method of detection detected a particular site of nodal recurrence better any other site (data not shown). In the seven patients who had nodal recurrence detected by US, the disease was node field recurrence only in five. There was no evidence of any site being more reliable for early detection by US (data not shown). Of all 24 patients found to have node field recurrence, it was node field recurrence only in 12 (5%); the remaining 12 patients (5%) showing concurrent disease at other sites. Of those who first recurred in the draining node field, 19 patients had more than one scan performed within a median of 18 days of the nodal recurrence. All nodal recurrences detected by US also were evident on PET/CT and all but one on CT. In one (0.5%) of the 19 patients, the nodal metastasis was not visible on all concurrent imaging modalities, as the lesion was visible on US and PET/CT, but not evident on CT.

### Pattern of Nodal Recurrence

As shown in Table 1, in 56 patients (25%) recurrence at any site except the node field and in 24 (11%) recurrence was in the SN field. In the patients who recurred in the SN field, half also had recurred at other sites at the time of recurrence detection (Table 3). These node field and other site(s) recurrences were “high-volume” disease, defined as at four or more sites in eight patients (4%).

**Table 3:** Description of concurrent node field and other site(s) disease at time of nodal recurrence as first recurrences in SN+ve patients

	Node field and other site(s), *n* = 12, (percentages of the whole cohort of SN+ patients, *n* = 225)
Burden of node field and other site(s) disease	
Oligometastastic disease*	4 (2%)
High-volume disease**	8 (4%)
Sites of disease***	
No evidence of distant metastasis	4 (2%)
Distant metastasis to lung	3 (1%)
Distant metastasis to non-CNS visceral sites	5 (2%)

*Oligometastastic disease defined as 1–3 site(s) of metastatic disease

**High-volume disease defined as ≥4 sites of disease

***M0, no evidence of distant metastasis; M1b, distant metastasis to lung; M1c, distant metastasis to non-CNS visceral sites

## Discussion

In this study of patients who were SN+ve and did not have a CLND, only a minority (5%) developed node field recurrence only in the SN+ve field, whereas others recurred both in the SN+ve node field and other sites, e.g., in-transit or distant metastasis (5%), or outside the SN+ve field (25%). For patients who recurred in the SN+ve field, this study demonstrated that all nodal recurrences were detectable on both US and PET/CT scans with a median scan interval of 18 days. Our data suggest that US may not need to be conducted concurrently if patients are having regular cross-sectional imaging.

Many of our results are consistent with previous studies. Montgomery et al. assessed 109 SN+ve patients in whom CLND was omitted, detecting 13 (12%) node field recurrences after a median follow-up of 15 months.^7^ In Montgomery’s study, where 57% received adjuvant therapy, only 24% experienced disease recurrence, but they had a significantly shorter median follow-up interval of only 15 months compared with 23 months in our study.

Bartlett et al. similarly examined 370 SN+ve patients in whom CLND was omitted. After a median follow-up of 33 months, 158 (43%) developed recurrences, of which 13% were node-only, 12% local, satellite and/or in-transit, 4% combined (local, satellite and/or in-transit with nodal involvement), and 14% systemic.^8^ Overall, the recurrence rate of 43 % in that study was higher than in our study where 36% recurred at any site, which may partly be explained by a much lower rate of adjuvant systemic therapy in Bartlett’s study (only 6% had adjuvant systemic therapy compared to 53% of our cohort). Another reason might be the longer duration of follow-up in their study compared with ours (median 33 vs. 23 months). In Bartlett’s study, imaging with CT, PET/CT, and/or US was done at the discretion of the attending physician. Most of the recurrences were detected by cross-sectional imaging (40%) with only 14% detected by node field US. This study did not, however, assess the detection of nodal recurrences by concurrent imaging results.

Node field recurrences in our cohort of SN+ve patients were detected at a median of 10 months after SNB, consistent with previous reports.^3–5,9^ The highest risk of recurrence in all previously reported studies and in the present study is within the first 2 years after initial melanoma diagnosis, justifying more intense surveillance during this period.^1,10–12^

The Breslow thickness, presence of ulceration and microsatellites, as well as increased mitotic rate are well-known risk factors for SN positivity.^13,14^ These factors also were associated with both nodal and other recurrences in our patient cohort (Table 1).

Detection of nodal recurrences will be determined by the test(s) that is performed and order hereof. The high rate of concurrent node field and other site(s) disease at the time of detection of nodal recurrences and/or recurrences at any site except node field in our cohort, highlights the importance of cross-sectional imaging in this patient group. Given that both PET/CT and US detected the nodal recurrences in all patients who underwent both scans, use of ultrasound in conjunction with cross-sectional imaging may be unnecessary if patients are followed-up by cross-sectional imaging. Ultrasound provides an imaging modality without radiation exposure, which is of particular importance in young patients with stage IIIA disease, who are at lower risk of recurrence. The type and frequency of surveillance imaging for SN+ve patients vary considerably, depending on clinician, imaging availability, and cost to the institution and patient and the optimal frequency and duration of imaging remain to be determined. Regardless of imaging modality, the potential risks of false-positive results, patient anxiety, and, for PET/CT and CT, radiation exposure must be considered.^10^

Inconsistency of type of imaging and intervals hereof is a limitation in our study. Only the scans performed within 2 months after detection of nodal recurrence in a SN+ve field were assessed in our study. Total number of scans, false-positive findings, and incidental findings were not addressed. Another limitation is the retrospective nature of the study and its limited size (225 patients), with only 24 recurrences in a node field. Furthermore, the follow-up interval was relatively short with a median of only 23 months, and there was uncertainty about blinding of the reporting sonographer, radiologist, and nuclear physician regarding reporting of concurrently or previously performed scans in the patients who developed SN+ve node field recurrence, which may have affected the reporting of recurrence detection.

Despite limitations, we report a cohort of SN+ve patients, treated in the current era, where CLND was omitted and where cross-sectional imaging was frequently used. This particularly distinguishes it from previous similar studies and increases the clinical applicability of our results.

## Conclusions

SN+ve patients for whom CLND was omitted are at risk of recurrences not only in the SN field but also at other sites (other locoregional recurrence and/or distant metastases). Moreover, nodal recurrences are often part of a multisite disease progression. These tend to occur with the highest incidence within the first 2 years after diagnosis and are best detected on cross-sectional imaging. Despite previously reported greater sensitivity of US for nodal metastasis detection, the higher rate of recurrences outside the node field and the comparable detection rate of nodal recurrences by cross-sectional imaging modalities suggests that routine, concurrent ultrasound surveillance may be unnecessary for SN+ve melanoma patients, irrespective of whether the patient receives adjuvant therapy, if regular cross-sectional imaging is performed, specifically PET/CT.

### Supplementary Information

Below is the link to the electronic supplementary material.

Supplementary file1 (DOCX 42 kb)
