# Supplementary material for: Role of Concurrent Ultrasound Surveillance of Sentinel Node-Positive Node Fields in Melanoma Patients Having Routine Cross-Sectional Imaging
Source: Ann Surg Oncol. 2023 Nov 15;31(3):1857–64. doi: 10.1245/s10434-023-14526-9 (PMC10838221; doi:10.1245/s10434-023-14526-9)
Supplement: Supplementary file 1 — Supplementary file1 (DOCX 42 kb) [file 10434_2023_14526_MOESM1_ESM.docx]

**Supplementary data**

*Size and extent of nodal recurrences*

The median short axis of the lymph node metastasis, documented in the imaging reports, was 9 mm, 15 mm, and 11 mm, when detected by US, CT, and PET/CT, respectively (supplementary table 4). In the 13 patients who underwent surgery, the median size (range) of the largest LN metastasis was 30 mm (30-30) if first detected by the patient, 14 mm (14-40) if first detected by US, 40 mm (24-50) if first detected by CT, and 35 mm (11-45) if first detected by PET/CT (Table 5). In these 13 patients, extracapsular extension was present in 10 patients (4%). Of the remaining three patients, one had no extracapsular extension, two had positive core biopsies with subsequent complete pathological responses to neoadjuvant therapy identified on TLND, and excision of the involved node (Table 6).

**Supplementary TABLE 4.** Short axis measurement of nodal recurrence assessed by the imaging modality by which it was detected.

|  | US (N = 6) | CT (N = 7) | PET/CT (N = 6) |
| --- | --- | --- | --- |
| **Smallest measurement of lymph node metastasis (short axis), mm** |  |  |  |
| Median (IQR) | 9.0 (7.2, 13.0) | 15.0 (11.0, 19.0) | 11.0 (8.5, 19.5) |
| Range | 4-20 | 9-36 | 6-26 |

Abbreviations: IQR, interquartile range

**Supplementary TABLE 5.** Maximum diameter of excised recurrent lymph node metastasis in the SN+ve field

|  | Patient (N = 1) | US (N = 3) | | CT (N = 5) | PET/CT (N = 4) |
| --- | --- | --- | --- | --- | --- |
| **Maximum diameter of excised node metastasis (mm, median (IQR), range) if detected by:** |  |  |  | |  |
| Median (IQR) | 30 (30, 30) | 14 (14, 27) | | 40 (25, 45) | 35 (22, 45) |
| Range | 30 - 30 | 14 - 40 | | 24 - 50 | 11 - 45 |

**Footnote Table 5.** Abbreviations: IQR, interquartile range; US, ultrasonography; CT, computed tomography; PET/CT, fluorodeoxyglucose positron emission tomography-CT.

**Supplementary TABLE 6.** Surgical treatment of nodal recurrence in the SN+ve field

|  | Underwent Surgical Treatment, n = 13, (percentages of the whole cohort of SN+ patients, n=225) |
| --- | --- |
| **Surgical treatment of nodal recurrence*** |  |
| Therapeutic lymph node dissection, TLND | 12 (5%) |
| Selective excision of nodes | 1 (0.5%) |
| If TLND or selective excision of nodes, number of involved nodes** |  |
| Median (IQR) | 2 (1.0, 10.0) |
| Range | 0 - 32 |
| **Maximum diameter of excised node metastasis (mm)** |  |
| Median (IQR) | 35.0 (24.8, 45.0) |
| Range | 14 - 50 |
| **Extracapsular extension in excised nodes** |  |
| Yes | 10 (4%) |
| No | 1 (0.5%) |
| No viable tumor | 2 (1%) |

**Footnote Table 6.** IQR, interquartile range; *Selective excision of nodes was performed in one patient with node field and other site(s) (incl nodal) recurrence. TLND was performed in 10 patients with node field recurrence only, and in 3 with node field and other site recurrences. One patient had anode excision, which revealed no metastatic cells but a complete pathological response to neoadjuvant therapy. One patient had a positive core biopsy with no positive nodes on CLND following neoadjuvant therapy.
